# Endocrine-Disrupting Chemicals and Their Effects in Pet Dogs and Cats: An Overview

**DOI:** 10.3390/ani13030378

**Published:** 2023-01-22

**Authors:** Paola Pocar, Valeria Grieco, Lucia Aidos, Vitaliano Borromeo

**Affiliations:** Department of Veterinary Medicine and Animal Sciences, University of Milan, Via dell’Università 6, 26900 Lodi, Italy

**Keywords:** endocrine-disrupting chemicals (EDCs), dog, cat, companion animals, reproduction, thyroid, human–animal interactions

## Abstract

**Simple Summary:**

Endocrine-disrupting chemicals (EDCs) are environmental pollutants with heterogeneous chemical structures and various sources that, once absorbed by the body, can interfere with endogenous hormonal actions. Extensive studies have been conducted correlating EDC exposure and human health, and interest in their effects on the health of domestic pets is increasingly becoming an issue of public concern. Dogs and cats can be exposed to EDCs in indoor and outdoor domestic environments through ingestion, inhalation, and transdermal absorption, with diet considered the primary source. Their exposure has been associated with several health disorders comparable to those described in humans, including thyroid and reproductive disorders, diabetes, heart and kidney diseases, and various types of cancer. The human–pet relationship implies sharing much of the same environment, thus including exposure to EDCs. Therefore, dogs and cats have been suggested as potential sentinels for human environmental exposure to contamination. It is becoming clear that exposure to EDCs is a matter of concern for pet health just as for humans, and the impact of this has been boosted by the growing attention to pet well-being. Any move towards a “risk-sharing” attitude in public health could, thus, benefit both humans and animals.

**Abstract:**

Over the past few decades, several pollutants classified as environmental endocrine-disrupting chemicals (EDCs) have become a matter of significant public health concern. Companion animals play a major role in human society, and pet ownership is substantially increasing worldwide. These intimate human–pet relationships imply sharing much of the same environment, thus including exposure to similar levels of EDCs in daily routine. Here, we review the current knowledge on the sources and routes of exposure to EDCs in domestic indoor and outdoor environments and discuss whether endocrine disruption is a health concern in pets. We summarize the phenomenon of endocrine disruption, providing examples of EDCs with a known impact on dog and cat health. Then, we propose an overview of the literature on the adverse effects of EDCs in domestic pets, with a special focus on the health of reproductive and thyroid systems. Finally, we explore the potential role of companion animals as unintentional sentinels of environmental exposure to EDCs and the implications for public health risk assessment in a “shared risk” scenario. Overall, this review supports the need for an integrated approach considering humans, animals, and the environment as a whole for a comprehensive assessment of the impact of EDCs on human and animal health.

## 1. Introduction

The Industrial Revolution started the contamination of the environment with various classes of pollutants, and this has dramatically increased, arousing substantial concern for environmental health globally. Over the past few decades, several pollutants classified as environmental have raised questions of significant public health concern. Endocrine-disrupting chemicals (EDCs) are among those posing significant threats for human and animal health.

According to the Endocrine Society, an EDC is defined as “an exogenous chemical or mixture of chemicals that interferes with any aspect of hormone action” [1]. From a mechanistic point of view, EDCs may mimic or interfere with endocrine homeostasis binding to hormone receptors on which they act either as agonists or antagonists. However, this basic knowledge of interference with hormones’ actions has expanded to include interactions with transcription factors, nuclear receptor coactivators, or even transgenerational effects by targeting germ cell lines [2]. Acting on endocrine systems through diverse mechanisms, EDCs can affect the reproductive and neuroendocrine systems, mammary gland development, and thyroid function [1]. They may also be involved in metabolic dysfunctions and obesity, as well as in the development and progression of endocrine-related cancers [3].

The molecules with endocrine-disrupting properties differ widely in chemical structure and include naturally occurring substances (e.g., phytoestrogens), synthetic molecules of anthropogenic origin (e.g., plasticizers, organohalogen compounds, industrial solvents, and pharmaceuticals), and heavy metals.

The relationships between EDCs and human health have been widely studied over the last 50 years, but interest in their effects on the health of domestic animals is much more recent and constantly expanding. This reflects people’s growing attention to pet health. Pet ownership is substantially increasing in most industrialized countries, and these animals are increasingly often considered “members of the family” [4]. Thus, any issue related to animal welfare is increasingly recognized, and care for companion animals is reaching high levels [5]. In addition, the close human–pet relationship implies sharing much of the environment, thus including exposure to similar levels of EDCs. Dogs and cats have, therefore, been suggested as unintentional sentinels of contamination in the human environment. 

Finally, in addition to the environment, food too is an important source of EDCs, and the safety of the huge global industrial pet food market is a potential emerging problem, arousing increasing concern not only for public opinion.

Here, we review the literature regarding exposure to EDCs in pet dogs and cats and the related adverse health effects and discuss the potential role of pets as environmental and health sentinels for human exposure.

## 2. Endocrine Disruptors with Known Risk for Domestic Pet Health

Hundreds of individual chemicals of anthropogenic or natural origin are known or suspected to act as EDCs. The list is long, and the chemical properties that dictate the sources of exposure and their environmental fates, vary widely (for a review: [3]). The present review focuses on classes of EDCs with known impacts on dogs’ and cats’ health. Table 1 summarizes the class, chemical structure, common uses, and health effects of the EDCs discussed.

### 2.1. Persistent Organic Pollutants

Persistent organic pollutants (POPs) are a wide group of organohalogen substances of anthropogenic origin that are resistant to environmental degradation and have very long half-lives (months to many years). Over recent decades, it has become clear that various POPs may act as EDCs in animals and humans [23]. These include several organochlorine pesticides (e.g., dichlorodiphenyltrichloroethane, (DDT)), industrial chemicals (e.g., polychlorinated biphenyls (PCBs), polybrominated diphenyl ethers (PBDEs), and perfluoroalkyl substances (PFAS)), as well as unintentional byproducts of industrial processes (e.g., dioxins). As a general rule, POPs share a number of common properties: (i) Resistance to environmental degradation and very long half-lives (months to many years). Their persistence and widespread application has led to POPs becoming ubiquitous in biotic and abiotic environments [24,25,26,27,28,29,30,31,32,33]. (ii) Lipophilic, accumulate in lipophilic tissues, and biomagnify through the food chain [34,35,36]. They have been detected in adipose tissue, serum, and breast milk samples collected worldwide of a variety of species, including humans [37,38,39,40,41,42,43,44,45,46,47]. Furthermore, their lipophilicity means they can easily cross the placenta, and preferential accumulation of selected POPs has been observed in offspring [48,49,50]. (iii) Volatile at certain temperatures and undergo long-range atmospheric transport, reaching places far from the site of their first use. In fact, many POPs can be measured in unspoiled environments far from the site of release due to the fact of long-distance transport through water and air currents and/or via migratory animals spending part of their life in polluted regions, which may become part of food webs in other uncontaminated areas [4].

#### 2.1.1. Polybrominated Diphenyl Ethers

Polybrominated diphenyl ethers are synthetic brominated compounds used as flame retardants in a range of polymer-based commercial and household products, such as textiles, furniture, and electronics, as well as construction materials to boost their flame resistance and meet increasingly strict fire safety standards [51]. The widespread use of PBDEs, starting in the 1970s, has resulted in persistent environmental contamination despite penta- and octa-brominated formulations being phased out in both the US and EU in 2004 [52,53].

The PBDEs’ main effects are endocrine disorders, with the thyroid gland as the primary target. This is due to the resemblance of the structures of PBDEs and their metabolites to thyroid hormones (THs), which leads to multiple methods of interference, such as competitive binding to serum transporters replacing thyroxine, reduction of protein activity involved in TH transport, and deregulation of TH metabolic enzymes [54,55]. It has been suggested that the adverse effects of PBDEs in neurobehavioral disorders and reproductive function may be linked to altered thyroid gland function due to the key role of THs in regulating multiple physiological functions. However, competitive inhibition of some PBDE congeners with the androgen receptor (AR) has been described both in vivo and in vitro, and this is likely to be at the basis of dysregulation of the development and function of androgen-dependent tissues [56].

#### 2.1.2. Polychlorinated Biphenyls

Polychlorinated biphenyls are synthetic organochlorine compounds that comprise 209 individual congeners identified according to the number and position of chlorine substitutions on the biphenyl backbone. Since the 1930s, PCBs have been mass produced worldwide for a variety of industrial purposes because of their nonflammability, chemical stability, and low electrical conductivity. The production and use of PCBs were banned in most industrialized countries by the end of the 1970s due to the growing concern regarding human health risks and environmental hazard. However, because of their long half-life and resistance to environmental degradation, they still linger ubiquitously in both terrestrial and aquatic environments. Furthermore, they are still gradually released worldwide into the environment because of improper disposal and incineration, leakage from landfills [57,58], and port activities [59,60].

From a mechanistic point of view, one of the most widely known classifications of the action of PCBs is based on physical structure and likeness to dioxin. Coplanar PCBs may exert dioxin-like effects through interaction with the aryl hydrocarbon receptor (AhR) [61] and are recognized as the most toxic congeners [62]. Noncoplanar PCBs make up a more varied group of congeners that are considered nondioxin-like because they have little or no activity at the AhR [63]. 

Related to the different degrees and patterns of chlorination, PCB congeners alter the activity of a panel of cytochrome P450 isoenzymes responsible for phase 1 activation of various drugs, carcinogens, and steroids [64,65] and may have carcinogenic, neurotoxic, and endocrine-disrupting activity. Their endocrine-disrupting effects mainly involve thyroid function through interference with the TH receptor [66] and reproductive function, exerting estrogenic, anti-estrogenic or anti-androgenic activity depending on the congener [67].

#### 2.1.3. Per- and Polyfluoroalkyl Substances

Per- and polyfluoroalkyl substances are a family of more than 3000 structures of highly fluorinated substances, including perfluorooctane sulfonate (PFOS), perfluorooctanoic acid (PFOA), and perfluorohexane sulfonate (PFHxS). PFAS can serve as surfactants, friction reducers, and water/dirt/oil repellants [68,69] and are, therefore, commonly used in the production of various everyday objects, such as food containers, kitchenware, clothes cleaning products, and electronic elements [70]. 

As regards their endocrine-disrupting activity, PFAS can potentially directly or indirectly impair the normal function of the reproductive and thyroid systems. However, there are still gaps in the literature regarding the mechanisms of action. PFAS may alter steroid hormone production or secretion, including that of estradiol, progesterone, and testosterone, and interfere with the gonadotropin endocrine balance [71,72,73]. In addition, PFAS can alter prolactin and human chorionic gonadotropin levels [74]. Finally, PFAS also act on normal thyroid function by dysregulating thyroid-stimulating hormone, triiodothyronine, and/or thyroxine levels [75,76].

### 2.2. Plasticizers

Phthalates and bisphenol A (BPA) are plasticizers that provide shape and flexibility to plastic products [77,78]. Phthalates are diesters of phthalic acid that are used as plasticizers in polyvinyl chloride products to make flexible plastics for building materials, medical devices, and food processing or packaging, or as solvents, fixatives, and adhesives in personal care products and cosmetics. BPA is a phenolic chemical that has been used for over 50 years in the manufacture of polycarbonate plastics and epoxy resins for consumer and food product packaging, including canned foods. These chemicals may leach out of the plastic—especially when containing warm food and drinks—into the food chain, causing toxic effects. Unlike POPs, plasticizers are not lipophilic and do not accumulate to any substantial degree in the body. They have a very short half-life (hours), are readily decomposed in the environment, and rapidly metabolized in the body [4]. Exposure to plasticizers varies, but since they are widely used in mass consumption products and the food industry, it occurs on a more or less daily basis. In humans, the majority of people tested have measurable levels of metabolites in their urine. Furthermore, both human and animal studies have demonstrated that phthalates and BPA cross the placenta, and metabolites are detectable in breast milk [79,80], posing a significant risk for developing organisms. In particular, organs that depend on a constant influx of hormones for proper functioning, such as the gonads, are especially vulnerable to the endocrine disruptor effects of plasticizers, predominantly during windows of susceptibility, such as fetal development, infancy, and puberty. Maternal exposure to phthalates and BPA has been associated with epigenetic reprogramming in gametes and early embryos, with the effects manifesting later in life, increasing the risk of disease [81,82,83].

In this context, prenatal exposure to phthalates promotes the transgenerational inheritance of both female and male reproductive dysfunctions [84,85]. This constant exposure, rapid metabolism, and the fact that the metabolites themselves have endocrine-disrupting activity [86,87,88] poses a further challenge for the assessment of phthalate and BPA endocrine-disrupting activity and can lead to underestimation unless the correct timing and biomarkers are used for analysis.

Much of the reported endocrine disruptor activity for phthalates and BPA has been related to their ability to interfere with estrogens, androgens, and THs; however, the mechanisms still have to be fully elucidated. As an example, the phenolic groupings in the BPA structure determine its ability to bind to estrogen receptors (ERs) and stimulate estrogen-dependent gene expression [89,90]. This can also interfere with estrogen’s physiological activity through membrane estrogen receptors and nongenomic mechanisms [91]. In addition to its estrogenic activity, BPA can bind to the AR and result in anti-androgenic responses [92]. Phthalates, too, can bind to both estrogen and AR and stimulate or inhibit ERs, but they have only inhibitory effects on ARs [93].

Both BPA and phthalates can significantly affect thyroid function [90,94]. However, both positive and negative correlations have been described between the urinary or serum levels of these classes of plasticizers and thyroid function [95,96,97]. Therefore, hypothetically plasticizers may behave as both a thyroid receptor (TR) agonist and a TR antagonist [98] or may also interfere with TH action by a nongenomic mechanism [99].

### 2.3. Natural Occurring Xeno-Estrogens

Chemicals structurally and/or functionally similar to mammalian estrogens and their active metabolites are produced naturally by many plants (phytoestrogens) and fungi (mycoestrogens). Phytoestrogens are secondary plant metabolites and have been classified into three major classes: isoflavones, coumestans, and lignans [100]. Mycoestrogens are natural estrogens produced by fungi and are thought to be harmful to animals when consumed in contaminated feed. 

The main mycoestrogen that can potentially occur in food is zearalenone (ZEA), a potent xenoestrogen produced by some Fusarium and Gibberella species. ZEA is heat-stable and is found worldwide in many cereals, such as maize, barley, oats, wheat, rice, and sorghum [101]. Soy is the richest source of phytoestrogens, while cereals and grains are most likely the main sources of mycoestrogen contamination [102]. 

Xenoestrogens (XEs) can exert wide ranging estrogenic effects by mimicking or blocking endogenous hormones and altering the feedback loops in the brain, pituitary, gonads, and thyroid. XEs were originally thought to act solely on nuclear estrogen receptors (nERs). Recent research has shown, however, that XEs can also have estrogenic effects through non-nuclear receptors, transcriptional coregulators, and enzymatic pathways [103].

## 3. Sources and Routes of Exposure to EDCs in Pets

**Figure 1:**
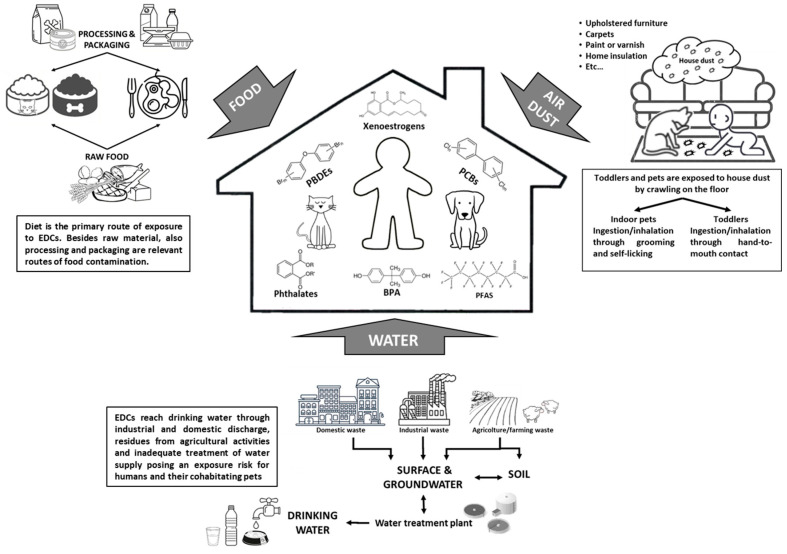
Routes of domestic pets’ exposure to EDCs – a shared risk with humans.

Dogs and cats can be exposed to EDCs in indoors and outdoors, in relation to the owner’s lifestyle, and both their living environment and dietary habits can contribute to the total body burden of EDCs through varying routes: ingestion, transdermal, and inhalation [104,105,106,107] (Figure 1). Oral ingestion is considered the main route of exposure to many environmental chemicals [104,108,109], with industrially formulated food the main source for pets [110].

Several EDCs were detected in both dry and wet pet food [7], and this is of particular concern considering that the market for pet food is constantly growing, with sales in 2021 reaching approximately 10 million tons in the USA [111] and 8.5 million tons in the EU [112]. A recent study in France reported contamination of industrially formulated dry cat food with phthalates (2292 ng g^−1^ for total phthalates), with diisononyl phthalate and di(2-ethylhexyl) phthalate (DEHP) as the prevailing compounds; with PCBs (1.7 ng g^−1^ for total PCBs), with PCBs 153 and 42 prevailing; and with PBDEs (0.088 ng g^−1^ for total PBDEs), with PBDE47 and then PBDE153/154 as the main representatives [113]. Similar concentrations of total PCBs were found in cat food in Japan [114] and Spain [115] and of phthalates in Italy [116]. The mean concentration of PBDEs (2.6 μg kg^−1^) was found in cat food in the USA [117] and might be explained by the use of penta-BDE commercial mixtures in that country as flame retardant in furniture, baby products, and carpet padding foam [118]. 

Levels of BPA are measurable in most dog and cat canned foods, and studies confirmed that the BPA in food originated from the can’s coating [119,120]. The BPA levels have ranged from 13 to 136 ng g^−1^ for canned cat food and 11 to 206 ng g^−1^ for dog food [119]. 

Diet is also the most important route of exposure to xenoestrogens in pets. Soy, which is rich in phytoestrogens, is a common pet food ingredient [102,121], and cereals and grains are a major source of mycotoxin contamination. It is commonly known that dry dog food contains larger amounts of cereals than wet dog food, and some studies, in fact, proved that dry dog food is contaminated with mycotoxins, including ZEA, at higher levels and frequency than wet dog food [122,123].

In addition to diet, other important routes of EDC ingestion in pets include chewing and mouthing objects. For example, phthalate and BPA present in dog chewing toys and bumpers can leach into saliva contributing to the body burden in pet dogs [124]. Furthermore, it has been observed that in addition to absorption through the gastrointestinal tract, BPA can also enter the body by direct absorption through the oral mucosa [125]. In humans, chewing and mouthing behaviors are largely confined to toddlers and have been acknowledged as sources of oral exposure to chemicals in toys, other children’s products, and household dust [126].

Intergovernmental organizations (such as the EU), countries, and some local governments regulate the chemicals in children’s and other consumer products in order to minimize the exposure of infants and children to chemicals that may pose health risks [127,128]. In contrast, only very general regulation to prevent the use of hazardous substances are defined by the Consumer Product Safety Commission concerning the production of pet chewing or mouthing objects [124]. It is noteworthy that amounts of BPA and phthalates leaching from dog toys showed similar levels to those measured in children’s toys before regulatory restrictions entered into force [124].

Another significant exposure route to different EDCs is house dust, especially for indoor-living cats because of their intense licking and grooming behavior [8,9,129]. Accordingly, serum PBDE concentrations in client-owned outdoor cats were significantly lower than in indoor cats, suggesting that house dust is a primary route of exposure to PBDEs in household cats [8,9]. It has also been recently confirmed that dust is an important exposure pathway to PFASs for cats, and the levels are similar in humans and pet cats [15,16,130].

While much has been written of the effects of EDCs from oral exposure, research is increasingly documenting their presence in air, which opens a debate on the risk from inhalation as a route of exposure. EDCs have been found in both outdoor and indoor air as volatile organic compounds (VOCs) or semi-volatile organic compounds (SVOCs) in the gas phase or attached to particulate matter (PM2.5 and PM10) [131,132]. In outdoor air, EDCs derive from agricultural and industrial activities, waste incineration, and from petrol and diesel fumes [131]. Therefore, the type and quantity of pollutants vary according to geographical location, urban versus rural sites, and with climate variations [133,134]. Indoor air, although supplied by outdoor air, can be additionally polluted by domestic activities, such as heating, cooking, tobacco smoking, and usage of chemical-based consumer products, especially from those in aerosol formats. The pollutant indoor air burden is further influenced in the modern world through reduced ventilation and dependence on air conditioning systems [132]. Many EDCs are indeed measurable often at higher levels in indoor than in outdoor air [135,136], indicating the indoor air microenvironment as the greatest inhalation exposure to EDCs for both humans and co-inhabitants companion animals [137]. The sources, origins, and partitioning of EDCs in air are reported in Table 2.

Despite the considerable body of evidence documenting the presence of EDCs in both outdoor and indoor air, many questions are still open, for example, the relative importance of the inhalation of EDCs in the gaseous versus PM format and the role of PM of different sizes as vehicles. It has also been suggested that for some semi-volatile EDCs, dermal uptake from air may be greater than uptake from inhalation [151].

## 4. Health Effects of Exposure to EDCs in Domestic Pets

A variety of EDCs (e.g., PCBs, organochlorine pesticides, dioxin-related compounds, phthalates, BPA, PBDEs, and PFASs) have been reported in cat and dog tissues, including genitals, adipose tissue, blood, urine, and fur [16,108,109,114,152,153]. However, in comparison with the large number of studies regarding EDCs and the health status of humans and wildlife, published studies on EDCs’ adverse effects in cats and dogs are scarce. Nevertheless, it is now recognized that concurrent with elevated exposure to environmental chemicals, there has been a steady increase in pet diseases that may be related to the effects of pollutants, such as cancer, thyroid disorders, diabetes, heart diseases, kidney diseases, and reproductive failure [7,154,155,156].

Here, we provide an overview of the current knowledge on EDC-related adverse health effects in domestic pets, focusing on the accumulating data linking exposure to EDCs to reproductive disorders and disturbed thyroid homeostasis.

### 4.1. Reproductive Disorders

In humans, reports of declining sperm counts over the last 50 years, together with epidemiological studies of the increased incidences of testicular cancer and genital tract abnormalities, strongly suggest an environmental adverse effect on male reproduction, with exposure to EDCs the most likely cause [157,158,159]. Specifically, there have been significant increases in cryptorchidism and hypospadias, which present at birth, and poor semen quality and testicular germ cell cancer (e.g., seminomas and their precursors and carcinoma in situ lesions), which manifest in young adulthood [160,161]. These changes all seem related and have been grouped under the common name of testicular dysgenesis syndrome (TDS) [162]. TDS has also been reported in various wildlife species, including fish, reptiles, birds, and mammals [163,164], suggesting that environmental factors might play a role in its pathogenesis. These disorders may have a common origin in fetal life and result from the disruption of embryonic programming of gonad development [162,165]. Thus, in utero exposure to EDCs with estrogen-like or anti-androgenic activities (e.g., phthalates, bisphenol A, PCBs, and some pesticides) have been both epidemiologically and experimentally linked to TDS lesions in several species [166,167,168,169,170], likely through interference with the physiological secretion of testosterone by fetal Leydig cells [167]. Recently, it has been suggested that developmental exposure to EDCs may lead to TDS also in male dogs. Exposure to DEHP or polychlorinated biphenyl 153 (PCB 153) increased cryptorchidism and affected sperm quality [7,10]. In addition, exposure to PBDE congeners was negatively correlated with the Sertoli cell number and male germ cell proliferative activity [6].

Finally, over the last 50 years, in parallel to what has been seen in humans, the incidence of canine testicular cancer has risen significantly [11,12]. Seminiferous tubule abnormalities and testicular germ cell neoplasia in situ comparable to human TDS have also been described in dogs [12]. These observations strongly suggest that exposure to EDCs may lead to TDS in dogs.

Reduced sperm counts have been widely used as an index of mammalian male subfertility. Meta-analytical studies indicate a 50% global reduction from 1938 to 2011 in sperm counts in humans [171,172,173]. Interestingly, the trend of the worsening of human semen quality over time is similar in dogs that live in a human household, where a 30% decline in sperm motility was observed over a 26 year period [7]. One can, therefore, hypothesize that temporal trends in semen quality in humans and dogs may be due to the shared environmental factors. Declining sperm quality has been linked with exposure to anthropogenic chemicals that have endocrine-disrupting activity [170], and a number of studies have shown that EDCs are present in adult testes and semen in various species, including humans and dogs, suggesting that these chemicals have a direct acute effect on sperm [7,174,175]. In dogs’ testes and ejaculate, DEHP and several PCB and PBDE congeners have been reported in a range of concentrations that have been demonstrated to perturb sperm viability, motility, and DNA integrity [7]. Interestingly, the same chemicals have been reported in different brands of commercial dog foods, suggesting food as a source of exposure [7].

In comparison with the large number of studies regarding EDCs’ effects on male reproductive health, very few studies have investigated their adverse effects in female reproduction, especially in pets. Bitches are monoestrous and fairly often suffer reproductive tract disorders, such as prolonged estrous, no estrous, or ovarian cysts [176]. It is believed that the specific hormonal regulation of reproductive processes in bitches, which involves long progesterone and prolactin cycles and high sensitivity to endogenous and exogenous estrogens, is believed to have an important role in the etiopathogenesis of those disorders [177,178]. Experimental studies showed that ZEA, a mycoestrogen often found in pet food [179,180,181], affects female reproductive organs [19,182]. Changes in bitches’ reproductive systems were mainly in the ovaries and the uterus, including the degeneration of cells, inhibition of biological activity in the ovaries, and edema and extravasations in the uterus [18]. Exposure to low doses of ZEA has been linked with permanent uterine dysfunction, frequently leading to the spontaneous endometritis–pyometra complex [20,21,22].

Recently, Sumner et al. [183] reported known environmental toxicants, including several PCB and PBDE congeners, present in dog ovary, in some cases at higher concentrations than in the testis. Both PCBs and PBDEs have been measured in the milk of nursing bitches. The transfer of lipophilic compounds into the milk is a known route by which lactating animals reduce the load of chemicals in their adipose tissue [184]. At the same time, however, the primary source of nutrition for the neonate becomes a source of exposure to the “maternal legacy” of lipophilic chemical contaminants and may lead to perturbation of reproductive development in pups. It was reported that the litters of maternal Arctic sled dogs fed seal blubber as a source of environmental toxicants presented a skewed sex ratio in favor of females [185]. 

A relationship between the feminization of dog litters and exposure to endocrine-active contaminants was also suggested by Lea et al. [7]. Investigating the outcome of a breeding program of assistance dogs, they reported a correlation between the altered sex ratio in litters in favor of females and the temporal decline in semen quality in stud dogs related to exposure to chemical contaminants.

### 4.2. Thyroid Disorders

The World Health Organization has reported that thyroid disorders (hypo- and hyperthyroidism) are among the most prevalent endocrinopathies in humans as well as in domestic pets [23,186,187]. A number of environmental contaminants disrupt TH signaling and homeostasis at numerous levels of hormonal action [188,189]. These include a range of organohalogen compounds, such as PCBs, organochlorine pesticides (e.g., DTT), PBDEs and PFAS, and the plasticizer BPA [190,191,192,193]. 

Feline hyperthyroidism (FH) is reported as one of the most common endocrine disorder in cats, especially in middle-aged and elderly subjects [194]. The frequency of the diagnosis of FH has increased significantly since 1979 when it was first described as a dis-tinct disease [7,195,196]. To date, it affects over 10% of older cats worldwide; though, the prevalence of FH varies significantly by geographical region [194,197]. While the pathology of FH has been exhaustively described [154,198,199,200], its etiology is still to be clarified.

Recently, it has been suggested that exposure to environmental thyroid-disrupting chemicals may contribute to the etiopathogenesis of the disease. For example, it has been observed that elevated total PBDE concentrations in home dust directly correlated with the incidence of hyperthyroidisms in indoor cats [8]. In addition, the mix of PBDE congeners in the serum of indoor cats was directly correlated with matched house dust samples [9]. Other compounds that can strongly disrupt thyroid function in cats are the per- and polyfluoroalkyl substances, which are ubiquitous in indoor environments. Recent studies indicate that serum from hyperthyroid cats had higher PFAS levels than hyperthyroid cats [15,16]. The association of serum levels of PFAS with the dust concentrations in cats’ homes confirms that dust is an important exposure pathway in this species [130]. 

In addition to the environment, food may be a source of exposure for cats to PBDEs, PCBs, and BPA, which have all been detected in cat food [104,119,120]. One large case-control study reported an association between hyperthyroidism and cats fed from “pop-top” cans where BPA was used to line the pop-top lids [17]. PCB and PBDE derivatives were also found in fish/seafood-flavored canned cat food with the types and amounts of chemicals and their metabolites consistent with those found in the fish used as raw material [107,201]. A questionnaire-based case-control study in the UK found that one of the risk factors for hyperthyroidism in cats was fish feed, which can contain high concentrations of POPs [193,202]. 

Although studies suggest that the etiopathogenesis of FH is presumably multifactorial and includes a combination of genetic, nutritional, and environmental factors rather than any single etiological agent, the literature strongly suggests that EDCs may play a role in its development by interacting synergistically with other factors in FH-prone individuals.

The effects of EDCs on thyroid homeostasis in domestic pets may be species-specific. Indeed, whereas in cats a positive correlation has been observed between hyperthyroidism and EDC exposure, in dogs thyroid disease typically presents as hypothyroidism, and studies showed a tendency to higher POP concentrations in hypothyroxinemic than in euthyroxinemic dogs [13,14]. 

Species-specific effects on TH may be related to different detoxification metabolisms in dogs and cats. PCBs are mainly metabolized in the liver to hydroxylated PCBs (OH-PCBs) during phase I metabolism. Some OH-PCBs with a hydroxyl group in the para-position with an adjacent chlorine atom are of particular concern on account of their structural similarity to TH. In particular, competitive binding of OH-PCBs to the TH transport protein transthyretin (TTR) in blood [203,204], may lead to the disruption of TH homeostasis [205,206,207]. In PCB-exposed dogs, the effects on TH levels were mainly due to the enhanced TH excretion and competitive binding of T4-like OH-PCBs to TTR [14]. However, serum patterns of OH-PCBs differ significantly between cats and dogs, with higher chlorinated OH-PCBs (T4-like OH-PCB), the predominant OH-PCB congeners in dog serum, while cats have higher levels of lower chlorinated OH-PCBs [208]. This might be attributable to differences in the metabolic capacity for PCBs and in the activity of CYP2B-like enzymes [209,210]. The binding affinity of lower-chlorinated OH-PCBs to TTR is weak [211], which may account for the different sensitivities of dogs and cats to EDC-related thyroid disruption.

## 5. Pet Dogs and Cats as Sentinels for Human Exposure

Sentinel species can be defined as animals that can be used to measure the extent and the health consequences of exposure to environmental hazards when measurement in human is impractical or unethical. The use of nonhuman organisms as early warning systems for human health risk is not new. The miner’s canary used to monitor air quality in coal mines is perhaps the most widely known example. Since then, interest in using sentinel animals in the field of environmental monitoring has constantly grown, and there is increasing pressure to unravel the links among animals, humans, and the environment through a “shared risk” paradigm [212]. 

Various factors explain why companion animals offer advantages as sentinels for human exposure and health. While environment/health outcome links in humans may appear biologically plausible and common sensical, in practice they can be hard to prove. Obstacles include lifestyle choices and habits, such as mobility, heterogeneity, and diverse exposure histories. For example, the long latency of many chronic conditions means that some people may not be diagnosed with a particular disease when they have already left an environment that may mask clear causal links between the forces of environmental change and human risk. In contrast, pets tend to be less mobile and may be exposed at higher levels to a given environmental hazard than their cohabiting humans, whose lifestyle choices may actively modify exposures.

The strength of epidemiological exposure studies in pets also lies in the relative freedom from concurrent exposures, bias due to the fact of confounding and, to some extent, exposure misclassification. In humans, the influence of cigarette smoking, alcohol, or occupational exposures may mask an effect of community exposure to environmental hazards [213]. Furthermore, pet dogs and cats have a shorter latency to the development of an environmentally induced health condition, on account of their smaller bodies and short lifespan [214,215], thus giving more insight into cause–effect relationships.

A particular advantage of pet cats as sentinels compared to other animals is that this species, ranging from indoor-only house pets to feral cats, occupies a number of different habitats. Sampling cats offers the combined advantage of monitoring both domestic species and urban wildlife species, rather than using one species as an indoor sentinel and another, with a different physiology, as an outdoor sentinel.

Pets may be particularly useful as sentinels for reproductive disorders. The routine surgical neutering of hundreds of thousands of dogs and cats worldwide provides easy access to surplus reproductive tissues, which can be used for research purposes. Furthermore, access to the controlled breeding populations of dogs routinely sampled for sperm quality may provide a cost-effective means of sperm analyses without the social implications that would accompany analogous human studies.

Finally, indoor pets may be particularly useful sentinels of chemical exposures for children. Pets live close to the ground, chewing on domestic objects, and licking and self-grooming—all habits similar to those of human toddlers, who spend a lot of time crawling on floors, with much hand-to-mouth activity [216,217]. As an example, it has been observed that increased floor contact time and grooming/mouthing behavior in both cats and young children (age 1.5 to 5 years) led to dust consumption up to seven-fold more than adult humans [218]. This correlates to POP serum levels in indoor cats as well as in children at significantly higher levels compared to their matching adults [219,220].

These considerations strongly suggest domestic pets as being suitable to serving as sentinels of human exposure to environmental pollutants and have, in fact, led numerous authors to use epidemiological data on pet environmental exposure as an indicator for human health risks [212,213,221,222,223,224,225]. However, the results have been variable and often contradictory. Differences in body size, diets, behavioral patterns, and/or xenobiotic metabolization systems might account for the range of findings. 

Some authors suggested that cats are adequate sentinels of human exposure to POPs [9,104,214], but others have not confirmed the role as sentinels of these contaminants in dogs [115,226]. Several authors reported that dogs show much lower levels of some POPs than other mammals (including cats and humans), even though dogs are exposed to higher dietary levels when fed commercial food [108,114,115,226,227]. Dogs, unlike the majority of mammals, efficiently metabolize and eliminate some POPs, which supports the hypothesis that this species would not be a good sentinel for human exposure and points to domestic cats as a better model to assess human exposure to these chemicals. However, the use of cats as sentinels for human exposure to POPs must be assessed carefully. Cats are hyper-carnivores and have a lower activity of certain cytochrome P450-enzymes involved in phase I and II reactions, limiting their ability to metabolize certain xenobiotics [228]. Accordingly, higher levels of various organohalogen compound residues were found in cats’ sera than in dogs [108,109] and humans [9,117,129].

Similarly, species-related differences have been observed for other classes of EDCs. For example, phthalate concentrations in cat urine were significantly higher than in dog samples from the same geographical area [215] due to the lower glucuronidation capacity in cats than in other species [153]. These observations strongly suggest that cats may tend to accumulate EDCs to a greater extent, because they are metabolically less equipped to degrade pollutants than dogs and humans [109,115,153], indicating that cats also do not fully represent human EDC exposure [229].

## 6. Conclusions

In summary, domestic pets are exposed to EDCs in indoor and outdoor environments and in their diets, with sources and exposure routes similar to humans. Furthermore, health effects comparable to those linked to human exposure to EDCs have been observed in cats and dogs, as well as the higher occurrence of various types of cancer, hyperthyroidism, and renal failure that have been associated with EDC contamination in diets.

The focus of the scientific community on the risks of EDCs still relies mainly on human medicine, and the effects on companion animals are often disregarded. However, EDC contamination is a global, ubiquitous public health problem and, on the basis of a “one health” concept, focus on companion animals should be considered as well.

Clearly, pet dogs and cats may hold out considerable potential as sentinels for human environmental exposure and point to a path for future research. However, there are still critical gaps in knowledge that need to be addressed. Although pet animals occupy the same environments as their owners and are expected to be exposed in broadly similar ways, their exposures are not quite the same, and quantitative information on the relative exposure risk is still incomplete. Formulations of pet and human foods are very different, and animals’ metabolism and pharmacokinetics for EDCs may differ significantly from humans, which also implies different relationships between exposure, tissue concentrations, and health outcomes. Moves towards a “shared risk” attitude in public health could, at least, partly overcome the current scientific barriers and provide tools to link human and animal environmental disease risks, with benefits for both human and animal health.

## Figures and Tables

**Table 1:** Class, chemical structure, common uses, and health effects of EDCs in domestic pets.

Class of Chemicals	Class	Chemical Structure	Reported Health Effects in Pets	References
PBDEs	Persistent organic pollutant	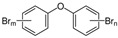	Decreased sperm quality and viability Testicular dysgenesis syndrome Hyperthyroidism (cats)	[6,7,8,9]
PCBs	Persistent organic pollutant	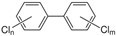	Decreased sperm count, quality, and viability Testicular dysgenesis syndrome Hyperthyroidism (cats) Hypothyroidism (dogs)	[7,10,11,12,13,14]
PFAS	Persistent organic pollutant	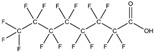	Hyperthyroidism (cats)	[15,16]
BPA	Plasticizer	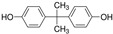	Hyperthyroidism (cats)	[17]
Phthalates	Plasticizer	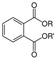	Decline in sperm quality Testicular dysgenesis syndrome	[7]
Zearalenone	Mycoestrogen	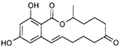	Degeneration of ovarian cells Endometritis–pyometra complex	[18,19,20,21,22]

**Table 2:** Sources and air partitioning of endocrine-disrupting chemicals in air.

Chemical	Source of Air Pollution	Air Partitioning	References
PBDEs	Soft furnishings	Distributed between the gas phase and PM, with the percentage of particle-bound PBDEs increasing with the increasing bromination number Higher levels in urban than rural areas Higher concentrations in indoor air than in outdoor air	[138,139]
PCBs	Hazardous waste sites; improper dumping of wastes; leaks or fires from electrical transformers or capacitors; waste incineration and open burning	Found in both gas and PM phases Higher levels in urban than rural areas Higher concentrations in indoor air than in outdoor air (10 to 100,000 fold)	[140,141,142]
PFAS	Stain resistance coatings in soft furnishings, fabrics, and floor waxing	More abundant in the PM than in the gas phase, with an increasing propensity for PM with the carbon chain length Indoor air concentrations half as high as outdoor air Highly concentrated in indoor dust	[143,144,145]
BPA	Plastic consumer goods, bottles, sports equipment, coating pipes and food cans, thermal paper, and burning of plastic materials	Found almost exclusively in PM10 Higher levels in urban than rural areas Higher atmospheric concentrations in cold season	[146,147,148]
Phthalates	Plastics consumer goods, personal care products, and air fresheners	Ubiquitous in indoor air More volatile phthalates (e.g., DBP) present in the gas phase and heavier phthalate (e.g., DEHP) predominant in PM Higher air concentrations at high ambient temperatures	[86,137,149,150]
